# Electrification of transportation means a lot more than a lot more electric vehicles

**DOI:** 10.1016/j.isci.2022.104376

**Published:** 2022-05-07

**Authors:** Michael A. Tamor, Ellen B. Stechel

**Affiliations:** 1 ASU School for the Future of Innovation in Society, Arizona State University, Tempe, AZ 85287-5603, USA; 2 ASU LightWorks and the School of Molecular Sciences, Arizona State University, Tempe, AZ 85287-5402, USA

**Keywords:** Energy policy, Energy sustainability, Energy transportation

## Abstract

A hidden barrier to the electrification of transportation is a lack of recognition of what it implies. Although the increasing popularity of battery electric vehicles (BEV) is heartening, the replacement of all personal vehicles with BEV would reduce US transportation emissions of CO_2_ by only about half. Aircraft and many ground vehicles are difficult or impossible to electrify. In meeting the “electrification challenge,” electricity is a medium for delivering fossil-carbon-free energy in a form suitable for each application whether mobile or stationary. This article synthesizes data from multiple sources to estimate how much biomass and GHG-free electricity will be needed to achieve carbon-neutrality in the US by 2050. Although subject to assumptions for growth and innovation, the resulting need for almost four times the electricity we use today and over 150 billion gallons per year of hydrocarbon fuel and feedstock are so striking as to provide meaningful policy guidance.

## Highlights

• We use the US EIA Annual Energy Outlook to construct a carbon-neutral economy in 2050
• Carbon neutrality requires nearly four times the electricity used today
• Electrochemical processes can bring biomass needs in line with expected availability
• With large dispatchable loads, the electric grid will operate differently from today’s

## Graphical abstract

**Figure:**
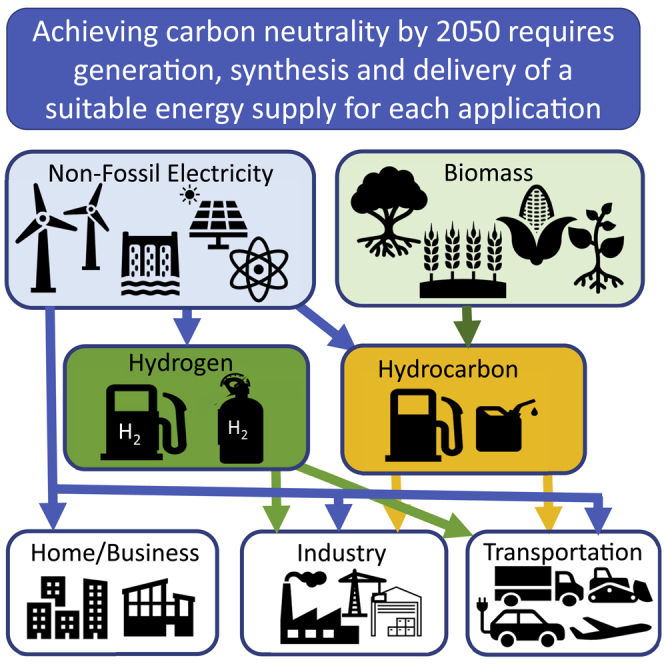

## Introduction

The commitment to achieve carbon-neutrality by the year 2050 as codified in COP21 Paris Climate Agreement and reaffirmed in COP26 Glasgow Climate Pact presents extraordinary technological, policy, and social challenges (IPCC, 2013; UN-FCCC, 2021a; UN-FCCC, 2021b). Today the stationary and mobile energy economies are essentially distinct: energy for transportation is dominated by liquid petroleum while stationary applications rely on a menu of other sources (coal, natural gas, nuclear, hydroelectric, wind, solar, and so forth). Electricity is expected to be the backbone of the carbon-neutral economy (Williams et al., 2021). Electricity can be generated from a variety of non-fossil energy sources and can be transmitted and converted to work with high efficiency, although it is at present difficult to store in quantity. [By “non-fossil” we mean without the release of fossil carbon into the atmosphere. This designation would include carbon-capture and storage (CCS) should it prove feasible and reliable.] In simple terms, all activities that can be effectively electrified will be, and those that cannot will be provisioned with non-fossil-based fuel. In turn, synthesis of non-fossil fuel is expected to be in part or entirely dependent on electricity (for example, H_2_ from electrolysis). Directly or indirectly, the economy will be largely electrified, and the stationary and mobile energy economies will rely on the same set of resources.

When planning a carbon-neutral energy economy it is just as important to consider what cannot be effectively electrified as it is to promote the conversion of activities that can be electrified. Electrification of the movement of people and goods presents exactly this challenge. First, because mobile objects usually carry their energy supply with them, energy density by both mass and volume of the storage medium is paramount. Although the present state of battery technology will serve the needs of most personal light-duty vehicles, there will remain many applications incompatible with range limitations, recharging times, or other logistic barriers. Second, the split between “direct” electrification using storage batteries or mobile power transfer and “indirect” electrification via fuel synthesis has a large effect on how much non-fossil electricity is needed to achieve carbon-neutrality. [For example, the electricity to make a unit of H_2_ by low-temperature electrolysis that would propel a fuel cell (FC) electric vehicle (FCEV) at a given distance would propel a BEV twice as far; a 50% loss from the generator to wheels.] In the public policy arena, the sheer scale of the energy transition is often underappreciated. In this article, we use a “spread sheet” model (more properly an inventory) of energy usage in a hypothetical 2050 economy to illustrate why we must undertake an unprecedented build-out of electricity generation, transmission, and storage, and how choices of transportation fuel will have a significant impact on the ultimate scale and operation of that new system.

Like many earlier studies, we use projections from the US Department of Energy (DoE) Annual Energy Outlook (AEO or Outlook) to construct a hypothetical carbon-neutral US economy in 2050 (EIA, 2021a). Jacobsen et *al*. (2015) used an earlier edition of the AEO to describe how energy from wind, water, and sunlight could replace all fossil fuels in the projected 2050 US economy. Williams et al. (2021) integrated the National Energy Modeling System (NEMS, on which the AEO is based) with models of the energy system to simulate multiple pathways to carbon neutrality. Both studies focus on the architecture of the future energy economy based on a series of assumptions for how electricity is substituted for fossil fuel. In both cases, road transportation is lumped into only two modes: light-duty (mainly passenger) and medium/heavy-duty (freight). The closest comparator to our work is the Net Zero America Project (NZA, Larson, et al., 2021). The NZA also begins with the AEO projections for sectoral energy use through 2050 and uses the EnergyPATHWAYS model and Regional Investment and Operations optimization tool (RIO, Evolved Energy Research, 2021) to find combinations of low-carbon technologies (including sequestration) that deliver that energy while minimizing societal cost. Although the NZA uses a more granular representation of road transportation, it does not capture the differences in usage between segments and the heterogeneity of usage within segments that limit the penetration of BEV. These “hard-to-electrify” surface transportation applications account for nearly half the demand for chemical fuel in a carbon-neutral economy.

To quantify the impacts of direct and indirect electrification and the choice of substitute fuel on the scale of the future energy system, we build a similar replacement model with a more granular segmentation that also includes non-transportation ground and military fuel needs. For a complete picture of the US energy economy and comparison to other studies, the model also includes residential, commercial, and industrial energy sectors. Based on well-understood technology and processes, we estimate the amount of electricity required to provide for three classes of usage: (1) the expected growth in electricity already in the AEO projection, (2) direct electrification of applications that would otherwise be served by fossil fuel, and (3) synthesis of H_2_ or hydrocarbon fuel for indirect electrification. Using published techno-economic studies of representative electrification pathways, renewable electricity, H_2_ synthesis by low-temperature electrolysis, and electro-biofuel synthesis, we then estimate the fuel cost for complete electrification (direct and indirect) of the 2050 economy.

We emphasize that in no way does this simple model compete with richer integrated macro-economic/energy system models designed to address many of the same issues. Owing to their complexity, it is difficult for readers to extract details of important assumptions that often appear in supplemental material and then sometimes in the form of references to other publications, and impossible to test variations to those assumptions. Instead, our model is intended as a tool to illustrate the immense scale of the electrification challenge and the impact of detailed assumptions of how much of which applications are electrified and how substitute fuels are produced. Despite its simplicity, the results of this “spreadsheet” model are in good agreement with published results from several integrated models. In the baseline carbon-neutral economy described in the next section, by 2050 we will need almost four times as much electricity as we use today supplemented by 1.04 billion tons of dry biomass. It also shows that the cost of decarbonization is modest and possibly negative on the scale of the entire economy. Although this model is highly simplified, it is logically defensible, completely transparent, and easy to test for sensitivities to key assumptions.

### Assumptions

#### The Annual Energy Outlook economic model

The AEO is based on output from the National Energy Modeling System (NEMS, 2019), which in turn is built around the Global Link Model, a proprietary global macroeconomic model developed and marketed by IHS Markit (IHS Markit, 2021). A characteristic of macroeconomic (ME) models =is that key inputs such as population, investment, and productivity change only gradually at rates based on historical trends. Projections in the AEO assume no CO_2_ emission regulations beyond those in force in 2020. The NEMS projects a 2050 economy that is a bigger version of todays with some shift in energy consumption between major economic sectors. Table 1 shows delivered energy in each of four economic sectors for 2020 and forecast in the AEO reference case for 2050. Like other integrated assessment models as exemplified by the Dynamic Integrated Climate Model (DICE) (Nordhaus, 2011), the NEMS can be used to capture the impact of carbon mitigation costs on economic growth. The simple substitution model described here does not capture this feedback; the carbon-neutral economy is assumed to be functionaly identical to that described in the AEO reference case and non-fossil energy is substituted for fossil fuel-derived energy without regard to cost. In principle this assumption is a serious drawback to the substitution approach. However, several studies using integrated models suggest that with anticipated cost reductions of known technologies, mitigation cost will be a sufficiently small fraction of GDP (less than 1%) such that this simplifying assumption is justified. In four energy transition scenarios for the U.S., Williams et al. (2021) found that the technical cost of carbon mitigation is between 0.38 and 0.89% of GDP and the resulting total GDP is essentially unchanged relative to the AEO reference case. Connolly and Mathiesen (2014a) found that a 100% renewable energy system for Ireland would be cost-neutral compared to a conventional system. Jacobsen et al. (2015) went even further in suggesting that when ancillary health and climate benefits of decarbonization are considered, a carbon-neutral energy system for the US would be cheaper than an expanded conventional energy system. In a more recent review, Koberle et al. (2021) make the same case that decarbonization will have a strong positive effect on output. Given the small or possibly negative net cost of decarbonization, the question of economic impact can be set aside, and a simple substitution model can be used to examine how much of what technologies will be needed to achieve our climate goals.

**Table 1:** AEO reference case forecast of energy delivered and fraction of energy delivered in each of the four major economic sectors

	2020	2050	2020	2050
Sector	QBTU	QBTU	%	%
Residential	11.36	12.03	16%	14%
Commercial	8.6	10.38	12%	12%
Industrial	25.47	34.16	36%	41%
Transportation	24.62	27.54	35%	33%
Total	70.05	84.11	100%	100%

Delivered energy does not include losses associated with the generation of electricity for delivery. Real GDP is forecast to grow from $18.2 trillion to $34.4 trillion by 2050, corresponding to a 38% reduction in energy intensity.

**Figure 1:**
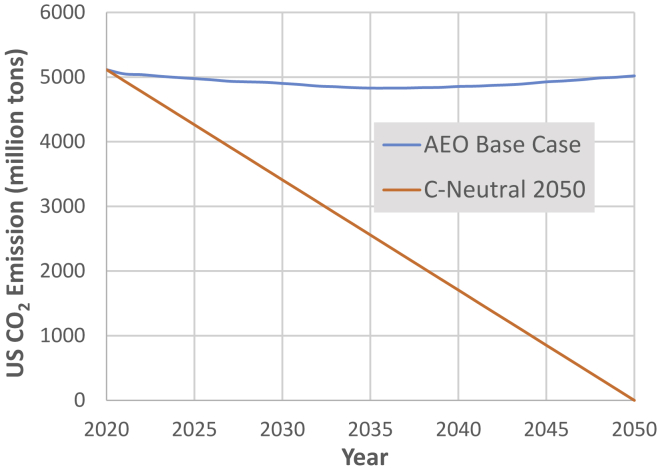
Projected US CO_2_ emissions through 2050 Upper curve: US CO_2_ emissions forecast in the AEO reference case (AEO, 2021). Lower curve: a hypothetical linear pathway to carbon neutrality in 2050.

The AEO report includes spreadsheets of projected energy consumption and fuel mix for 51 economic activities in the four economic sectors (Table 2). The trajectory of CO_2_ emissions forecast in the AEO reference case is shown in Figure 1. The slight decrease in emissions attributable to decreasing energy intensity of the economy is overwhelmed by overall growth leaving emissions in 2050 nearly the same as in 2020. Figure 1 also shows the hypothetical linear decrease in CO_2_ emissions reaching zero in 2050, as used by Williams et al. (2021) and Larson et al. (2021).

**Table 2:** Projected energy source and consumption for economic subsectors from the 2021 Annual Energy Outlook

QBTU	Electricty	Natural Gas	Petroleum	Coal
**Residential**				
Space Heating	0.60	3.18	0.48	
Space Cooling	1.42	0.06		
Water Heating	0.67	1.18	0.06	
Refrigeration	0.33			
Cooking	0.06	0.12	0.01	
Clothes Dryers	0.32	0.06		
Freezers	0.07			
Lighting	0.19			
Clothes Washers	0.05			
Dishwashers	0.04			
Televisions and Related Equipment	0.28			
Computers and Related Equipment	0.04			
Furnace Fans and Boiler Circulation Pumps	0.07			
Other Uses	2.48	0.22	0.14	
Residential Subtotal	6.62	4.82	0.69	
**Commercial**				
Space Heating	0.08	1.73	0.16	
Space Cooling	0.70	0.02	0.01	
Water Heating	0.02	0.71		
Ventilation	0.40			
Cooking	0.08	0.47		
Lighting	0.34			
Refrigeration	0.70			
Computing	0.35			
Office Equipment	0.90			
Other Uses	2.32	0.80	0.11	
Commercial Subtotals	5.89	3.73	0.28	
**Manufacturing**				
Food Products	0.38	0.95	0.02	0.12
Paper Products	0.12	0.44	0.01	0.06
Bulk Chemicals Process	0.42	3.89	0.34	0.05
Bulk Chemicals Feedstok		0.78	4.25	
Glass	0.05	0.13	0.00	
Cement and Lime	0.04	0.02	0.01	0.06
Iron and Steel	0.19	0.27	0.00	0.41
Aluminum	0.08	0.11	0.01	
Fabricated Metal Products	0.18	0.25	0.01	
Machinery	0.10	0.08	0.01	0.00
Computers and Electronics	0.15	0.08	0.00	
Transportation Equipment	0.18	0.21	0.01	0.00
Electrical Equipment	0.06	0.04	0.02	
Wood Products	0.08	0.08	0.02	0.00
Plastics	0.20	0.15	0.01	0.00
Balance of Manufacturing	0.51	1.29	0.12	0.08
Manufacturing Subtotal	2.74	8.78	4.84	0.78
**Nonmanufacturing Non-transportation**				
Agriculture	0.42	0.27	0.00	
Construction	0.27	0.02	0.00	
Mining	0.36	0.45	0.00	0.09
Nonmanufacturing Subtotal	1.05	0.74	0.00	
**Transportation**				
Automobiles	0.37		3.99	
Light Trucks (Class 1)			8.96	
Commercial Light Trucks (Class 2a)	0.01		1.02	
Motorcycles			0.01	
Buses - Transit	0.01	0.02	0.08	
Buses - Intercity	0.00	0.00	0.04	
Buses - School		0.00	0.10	
Freight Truck Light Medium (Class 2b)			0.93	
Freight Truck Medium (Class 3–6)			1.23	
Freight TruckLarge (Class 7–8)			3.61	
Aviation - General			0.22	
Aviation - Domestic Passenger			1.95	
Aviation - International Passenger			1.18	
Aviation - Dedicated Freight			0.75	
Maritime - Domestic Shipping		0.00	0.05	
Maritime - International Shipping			0.89	
Maritime - Recreational Boats			0.18	
Rail Freight		0.19	0.25	
Rail Intercity Passenger	0.00		0.01	
Rail Transit Passenger	0.02		0.00	
Rail Commuter Passenger	0.01		0.02	
Lubricants			0.12	
Pipeline Fuel Natural Gas			0.71	
Military Jet Fuel and Aviation Gasoline			0.40	
Military Residual Fuel Oil			0.02	
Military Distillates and Diesel			0.12	
Transportation Subtotals	0.42	0.22	26.85	
Industrial Nonmanufacturing - Agriculture			1.01	
Industrial Nonmanufacturing - Construction			1.25	
Industrial Nonmanufactruing - Mining			0.26	
Nonmanufacturing Transportaion Subtotal	0.00	0.00	2.51	
All Transportation Subtotal	0.42	0.22	29.36	
	**Electricty**	**Natural Gas**	**Petroleum**	**Coal**
Total Energy QBTU	16.72	18.29	35.17	0.78

Fossil energy sources are collapsed into solid (coal of all types), petroleum (distillates, gasoline, natural gasoline, and propane), and natural gas.

#### Carbon sequestration and the future of petroleum

An important simplification in our approach is the assumption that fossil fuel use will cease and supporting industries disappear by 2050, and achieving carbon neutrality will not require carbon sequestration. Given the interest in using carbon capture and storage (CCS) from stationary sources or from the atmosphere (direct air capture or DAC), or bio-energy with CCS to offset the use of fossil fuel in hard-to-electrify applications, it is necessary to justify this constraint in terms of plausibility, electricity needs, and cost. As the results of this study show (see Section Results), with an aggressive program of electrification, there is ample biomass to provide fuel for chemical feedstock and the remaining high heat and high energy density applications. Furthermore, carbon-efficient biofuel is slightly carbon negative. Conversion of one billion tons of biomass results in 160 million tons of char containing 125 million tons of carbon, equivalent to temporary sequestration of 350 million tons of CO_2_ (Lam et al., 2017). In terms of energy, the use of DAC and sequestration to offset emissions from fossil fuel use appears to be favorable; several studies suggest that as little as 4 kWh of electricity will be needed to recapture and store the CO_2_ released by burning one gallon of gasoline (Lackner, 2009, Realmonte et al., 2019); . This value is only 12% of the heating value of that gallon of fuel or 30% of the work from a vehicle engine using that fuel (see Section electrification pathways). However, DAC is capital intensive and cost estimates vary widely. Multiple studies (Lackner, 2009; Realmonte et al., 2019; Fashi et al., 2019) suggest a range of $30-$350/ton, which equates to $0.27 to $3.11 per gallon of fuel. This “carbon tax” offsets part or all of the cost differential between fossil petroleum fuel and the projected cost of biofuels (Das and Saffron, 2019). Finally, without energy-intensive conversion to a stable solid form, for example carbonate or synthetic anthracite, the permanence of any form of carbon sequestration on the required multi-millennial timescale is not yet assured.

#### Transportation energy

Transportation emissions are reduced by a combination of four actions: improving the efficiency of every mode, transitioning to non-fossil energy, shifting to less energy-intensive travel modes, and reducing the distances over which people and goods move. Efficiency improvements and a modest shift to electrification from a somewhat cleaner power grid are built into the AEO reference case. Some researchers advocate that a mass movement to high-density cities with public transit within walking distance of every home, job, and shop - and without automobiles - is essential to achieving our climate goals (Neuman and Kenworthy, 2015), and the New Urbanism movement promotes the benefits of higher density transit-ready development (New Urbanism, 2021). Other studies suggest that the energy benefits of densification are modest (Mindali et al., 2004). Given the lively public discussion of the energy and lifestyle benefits of re-urbanization, its omission here requires explanation.

##### Population density

**Figure 2:**
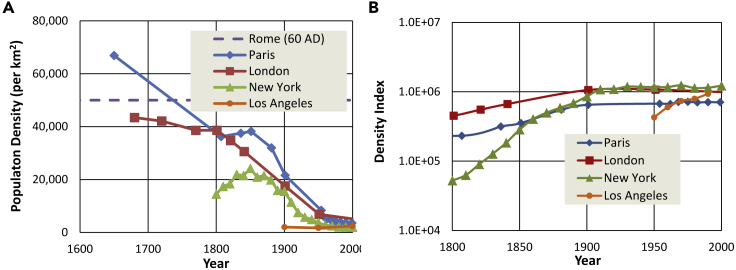
Historical labor market population density for four major metropolitan areas (A) Population density. (B) population density index, P/A^1/3^ ((Lincoln Institute of Land Policy, 2016).

While over 80% of the US population lives in what are defined as urban areas (US Census, 2010), only a small fraction lives in high-density areas that we associate with accessible public transportation. Labor market population density, the measure of density in the contiguous region in which residents live, work, and play in the US is quite low by global standards (Demographia, 2021). [The “labor market” is the region through which residents move irrespective of administrative boundaries, and so is most relevant to transportation issues.] Los Angeles, the densest major metropolitan area in the US, boasts only 2400 residents per km^2^ (slightly higher than greater New York) compared to London with 5700 residents per km^2^. Economically viable public transit requires high usage and commensurately high population density. In their guidelines for transit-oriented development, Cervero and Guerra (2011) recommend a minimum residential density of 6900 per km^2^, over three times the density of Los Angeles or New York. Even with a consensus for such a radical revision to the living conditions of most Americans, it would be wildly optimistic to assume that such a transition could be completed in just three decades. Furthermore, such a strategy seeks to reverse historical trends. Figure 2A illustrates that urban sprawl as indicated by a precipitous drop in population density even as the total population grows dates to the mid-nineteenth century long before automobiles appeared in cities (Angel et al., 2012). The great mass transit systems of London, Paris, and New York were launched when typical urban population densities were five to ten times those we see today. Even before the advent of the personal automobile, city dwellers were willing to exchange travel time and expense for more spacious living conditions. In fact, there is reason to believe that streetcars and later light rail systems were early facilitators of urban sprawl (Stewart, 2016).

Based on data from US cities between 1950 and 2000, Marshal (2006) proposed an empirical scaling relationship between urban population, P and land area, A, where the linear population density, P/A^1/2^ does not change as a city grows. The much longer timeline in the historical data of Figure 2A suggests a slightly different invariant density index of P/A^1/3^ shown in Figure 2B. The modified index is virtually constant over the last century in New York, London, and Paris, but still increasing gradually in significantly younger Los Angeles. This persistent scaling behavior suggests that powerful trends will continue to drive further decreases in density over the next few decades. Even if densification were possible, it cannot affect the distance that goods move between cities, factories, and ports, and so would have little impact on the large fraction of transportation energy (roughly 30%) consumed moving freight.

##### Mode shifting

With little scope to reduce passenger miles or ton miles (a metric of freight transport indicating the movement of one ton of freight a distance of one mile), we must consider the energy used instead. Table 3 compares the energy use per passenger-mile of transportation modes (Transportation Data Book, Davis and Boundy, 2019). The table shows that the in-use efficiency of the modern automobile is comparable to that of other transportation modes. In terms of GHG emissions, today there is little to choose between personal hybrid-electric cars, buses, and trains. In the cases where a direct comparison is available, direct electrification with today’s mix of generation reduces CO_2_ emissions by roughly 50%. For passengers and freight, the fuel is much more important than the mode.

**Table 3:** Comparison of per-mile and per-passenger-mile energy requirements of passenger transportation modes

Mode	Load Factor (person per vehicle or %)	BTU fuel per passenger mile	kWh electricity per passenger-mile	CO_2_ grams per passenger-mile
Car (on-road)	1.6	3034		214
Car (EPA, 2021 target)	1.6	2034		144
HEV (Fusion, Accord)	1.6	1653		117
BEV (midsize car)	1.6		0.16	62^c^
Personal Truck	1.8	3345		236
Bus				
Transit	10%	4025		298
Intercity	60%	477		35
Rail				
Intercity	50%^a^	1663		123
Transit	26%^b^		0.2	77^c^
Commuter	32%^a^	1643		122
Commercial Air	80%	2332		168

CO_2_ emissions from hydrocarbon fuels are computed using emissions coefficients from the U.S. Energy Information Agency (EIA, 2021c): 70.66 kg/MBTU for gasoline, 74.14 kg/MBTU for diesel fuel, and 72.2 kg/MBTU for jet fuel. CO_2_ emissions from electrified vehicles are computed using 2020 grid-average electricity generation. Upstream emissions from electric vehicles vary considerably with region, season, and time of day. The table illustrates the importance of load factor in efficient mass transportation.

a 90 seats/vehicle.

b 100 seats/vehicle.

c 385g/kWh.

##### Autonomous vehicles

In principle, autonomous vehicles (AV), can deliver significant energy savings by multiple means: elimination of inefficient driving habits, more efficient routing, connection to traffic management infrastructure, higher load factor via ride sharing, and ultimately vehicle weight reduction enabled by the elimination of collisions. Conversely, the newfound convenience of shared AV may result in a mode shift away from public transportation and a general increase in vehicle travel. Estimates of the energy impact of AV depend on the chosen scenario and so vary widely from roughly halving to doubling personal vehicle energy consumption (Stephens et al., 2016). Given this ambiguity, we do not include autonomous vehicles as a transportation option in this study.

#### Electrification pathways

Transportation is electrified via two pathways, direct and indirect. Direct electrification takes two forms: charging batteries in a BEV and delivering power through a wired or wireless connection above or below the roadway to a tethered electric vehicle (TEV). TEV can be equipped with batteries for short-range operation between loading points and electrified highways. The amount of petroleum fuel displaced by a given amount of electricity will vary with technology and application. For simplicity, we compare the fuel consumption of a modern HEV (50 miles per gallon) to the electricity consumption of a similar size BEV (0.25 kWh per mile) in which case one kilowatt hour (kWh) of electricity displaces 8520 BTU of fuel. This equates to 0.08 gallons of gasoline without ethanol, for which the lower heating value (LHV) is 116,090 BTU per gallon (AFDC, 2021). [Note that the LHV of finished gasoline varies somewhat with formulation and decreases with alcohol content.] This energy value corresponds to a net thermal efficiency of 40%, similar to the cycle-average thermal efficiencies of modern compound diesel truck engines (FutureTruck, 2015) and gasoline hybrid electric vehicles (Matsuo et al., 2016).

#### Carbon-neutrality model

The baseline carbon neutrality model assumes that by 2050 hydrogen will be produced by low-temperature electrolysis (e-H_2_) and delivered to the vehicle with a net efficiency of 70% (based on the lower heating value, LHV, of hydrogen) or 48 kWh/kg. In vehicles, we assume H_2_ is used in FCs with 60% cycle-averaged efficiency in cars and 50% efficiency in trucks. The lower value for heavy vehicles reflects typical operation at a much higher fraction of rated power (Lohse-Busch et al., 2020). No efficiency correction is made when substituting H_2_ for hydrocarbon fuel in aviation or for combustion heat in stationary industrial applications.

The model assumes that electro-biofuel (ebFuel) is used as a direct replacement for fossil petroleum as fuel and as a bulk chemical feedstock. Electro-biofuel is synthesized by pyrolysis followed by electro-catalysis of dry biomass (py-ECH). The resulting mix of hydrocarbons can be refined to make substitute gasoline and diesel products. The py-ECH process retains nearly twice the input carbon than does conventional fermentation and so makes much better use of limited biomass resources. In the process proposed by Lam et al. (2017), it takes 20 kWh of electricity and 6.6 kg of dry biomass to make one gallon of gasoline-equivalent fuel. [One gallon of gasoline equivalent (gge) fuel has the same lower heating value (116,090 BTU) as one gallon of conventional gasoline.] Within the limits of biomass availability, ebFuel is also used as feedstock for the bulk chemical industry. This substitution amounts to the assumption that carbon in bulk chemicals otherwise derived from fossil fuel, even when incorporated into durable products, is not permanently sequestered. A more sophisticated approach to the permanence of carbon sequestration in products and non-hydrocarbon substitutes should result in a reduced biomass demand for bulk chemicals and possibly justify the use of fossil carbon for products where sequestration is assured.

Direct air capture (DAC) of carbon dioxide followed by conversion to syngas (a mixture of carbon monoxide (CO) and H_2_) and then Fischer-Tropsch-like conversion to a familiar hydrocarbon fuel (electro-fuel, eFuel) requires no biomass but much more input energy. [Note that by 2050 there will be little or no CO_2_ emission from stationary sources suitable for carbon capture.] Because the technology for eFuel synthesis is far less advanced than that of H_2_ production and energy input can be a combination of electricity and heat, a single reliable value for electricity input per gallon-equivalent of fuel synthesized is not available. A minimum value can be derived from the direct synthesis reaction

(Equation 1) CO_2_ + 3H_2_ → -CH_2_- + 2H_2_O + 125 kJ.

Synthesis of one gallon of gasoline requires 1.2 kg of H_2_. In turn, synthesis of that 1.2 kg of H_2_ by low-temperature electrolysis at 80% energy efficiency requires 49 kWh of electricity, over twice that needed to synthesize the same amount of ebFuel. More realistic values can be inferred from recent techno-economic studies. Brynolf et al. (2017) find that the sensitivity of synthetic fuel cost to electricity cost is the same for multiple synthesis pathways corresponding to 63 kWh/gge. Kim et al. (2012) describe a family of sun-to-fuel processes designed around solar concentration systems used to drive chemical conversion, generate electricity, and provide heat for other process steps. For a mixed Fischer-Tropsch pathway with the separate thermochemical splitting of water and CO_2_, the overall process efficiency of solar energy to the heating value of fuel is 11.3%. If the same sunlight were used to generate electricity only at the 20% efficiency assumed in that work, 60 kWh of electricity could be generated for each gallon of fuel synthesized. Although an indirect comparison, this is in good agreement with that from Brynof et al. These estimates do not include the energy cost of collecting CO_2_ from the air. Although the theoretical minimum capture energy is 3 kWh per gallon of gasoline, techno-economic analyses of DAC vary widely from 4 kWh (Lackner, 2009; Realmonte et al., 2019) to as much as 30 kWh depending on the chosen process and system assumptions (Fasihi et al., 2019). As a baseline we assume that the lower value can be achieved by 2050, yielding a total need for 67 kWh of electricity for each gallon of eFuel synthesized.

Ammonia (NH_3_) produced from renewable hydrogen and atmospheric nitrogen via the Haber-Bosch or other synthetic processes can be used as a carrier for hydrogen or as a combustion fuel (Kobayashi et al. 2019; Valera-Medina et al., 2021). The use of ammonia as a hydrogen carrier in an e-H_2_ pathway does not alter the energy required to produce that hydrogen and energy losses in formation and dissociation must be compared in detail to those of conventional hydrogen storage and distribution. The same energy argument holds for ammonia as a combustion fuel. Any advantages of ammonia such as the ease of nitrogen capture must outweigh several deficits relative to synthetic hydrocarbon fuel; it is toxic, corrosive, must be stored under pressure (similar to propane), and has less than half the volumetric energy density. Although ammonia may emerge as an important renewable energy or energy carrier pathway, its inclusion would complicate this study without affecting its conclusions with regard to future electricity needs.

The ranking of the four electrification pathways in terms of electricity intensity is robust. Direct electrification suffers no conversion losses. Electro-biofuel derives a large fraction of its energy from biomass. On a heat value basis, H_2_ takes less energy to make than electro-fuel and is more efficiently used than hydrocarbon in the vehicle. Hydrocarbon electro-fuels from captured CO_2_ take the most energy to synthesize and are used less efficiently in the vehicle. High-temperature electrolysis would reduce electricity requirements of each of the three, fuel synthesis pathways, but would tend to increase the advantage of H_2_ over hydrocarbon electro-fuel. Carbon neutrality scenarios are generated by selecting a suitable non-fossil substitute fuel for each activity or vehicle application and summing the requisite electricity and biomass.

#### Light-duty vehicles

**Figure 3:**
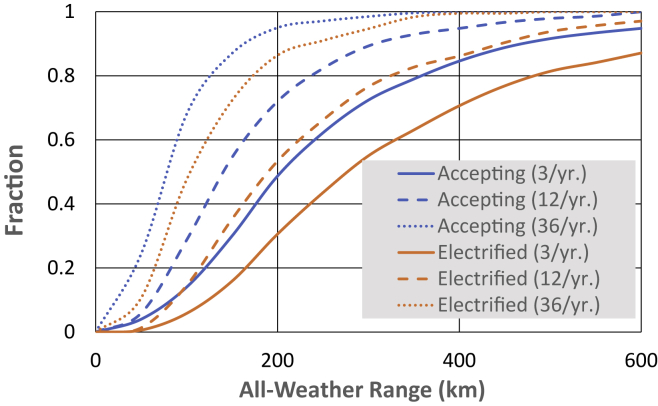
Acceptance electric vehicles as a function of range Blue curves: fraction of vehicles that may be replaced by BEV as a function of all-weather range for three levels of inconvenience of on-road fast-charging or finding alternative transportation (3, 12, and 36 days each year). Orange curves: the fraction of personal vehicle travel electrified as a function of range and inconvenience.

The AEO classifies vehicles up to 8500 lbs. gross vehicle weight (GVW) as “light-duty” vehicles (LDV). This definition of LDV includes Class 1 (up to 6000 lbs. GVW) vehicles in familiar household use (commuting, errands, and so forth) as well as the larger Class 2a trucks and vans often in full- or part-time commercial service. Personal vehicles are by far the easiest to electrify. Multiple studies have shown that BEVs will be acceptable in most households when three conditions are met: 1) the all-weather range exceeds roughly 500 km (300 miles), 2) convenient Level 2 overnight charging is provided at a home location for every BEV, and 3) convenient fast charging is available for occasional long journeys (Pearre et al., 2011; Tamor et al. 2015; Lin, 2020). Using the analytic method described by Tamor (2018), we can estimate the fraction of vehicles that might be conveniently replaced with a BEV as a function of range and a metric of inconvenience defined as the number of days per year requiring either a visit to a fast charger or finding another vehicle with sufficient range (Figure 3). For all-weather range of 500 km and insisting on just three or fewer days of inconvenience each year, 90% of conventional personal vehicles could be replaced thereby electrifying 80% of personal vehicle travel. If up to one visit to a fast charger per month would be acceptable, these replacement fractions rise to 98% of vehicles and 90% of travel. Keeping in mind that this acceptability threshold is a maximum and most users will experience less or even no inconvenience, the latter assumption is taken as the baseline for cars and personal-use trucks in this study

Plug-in hybrid-electric vehicles (PHEV) electrify the first portion of any journey (assuming it began with a full charge) but face no range limitations after their modest electric range is exhausted. In principle, PHEV could electrify a portion of personal travel where BEV are unacceptable. However, for a given electric range, the utility factor (the fraction of travel that is electrified) of PHEV in this subset of vehicles that drive the greatest distances will be much lower than for the population as a whole. Given the complexity of quantifying their limited contribution to electrification, PHEV are not included as an alternative here but will be the topic of a later study.

It is more difficult to account for the larger Class 1 and Class 2a light-duty vehicles in commercial use. The Transportation Data Book reports that 86% of light-duty trucks are for personal use, but personal use accounts for only 81% of light-duty truck travel and 78% of light-duty truck fuel consumption (Davis and Boundy, 2021, Table A17). This statistic implies that average fuel consumption and presumably average driving distance, in non-personal use is nearly twice that in personal use. For the purpose of estimating acceptance, doubling of driving distance is equivalent to halving the range scale in Figure 3. Under the same assumption for acceptability, this result translates to the electrification of 65% of non-personal light truck travel. If 90% of personal-use truck travel is electrified, similar to the fraction for cars, 84% of all light-duty truck travel (including personal and non-personal use) is electrified in the base case.

#### Commercial light and medium trucks

The AEO defines commercial light trucks (CLT) as vehicles between 8500 and 10,000 pounds gross vehicle weight (GVW). The diversity of usage of light and medium trucks (route delivery, remote industrial and agricultural service, power for on-board equipment, and so forth) suggests that a large fraction needs more energy than available batteries could provide or operate far from the fast-charging network that serves lighter vehicles. For the baseline model, we (arbitrarily for argument’s sake) assume that 50% of commercial LDV are battery electric.

#### Heavy trucks

Fuel consumption in freight transport is dominated by the long-haul operation of Class 7 and Class 8 trucks. These very long-range vehicles (1000–2500 km) operate mainly on the interstate highway system and are fueled at a network of several hundred truck plazas (compared to roughly 100,000 retail gas stations in the US). This tremendous range is not achievable with current battery technology, and we cannot assume the requisite breakthroughs in energy storage. This leaves two options: 1) provision of a chemical fuel - H_2_ for FCs or hydrocarbon for familiar diesel engines - or 2) provision of overhead or in-road power delivery for tethered operation on the highway. Both are approaching commercialization (Nikola, 2021; Siemens, 2021) and the trade-off between the large capital expense of an extensive in-road power system and higher efficiency of highway electrification is not yet clear. Several studies suggest that TEV can be more cost-effective than BEV (Connolly, 2017; Domingues-Olivaria et al., 2018). Here, highway electrification is assumed for the baseline case, but the impact of using non-fossil H_2_ or hydrocarbon must be considered.

#### Buses

Transit and school buses are assumed to be 100% electric. Intercity buses are treated as heavy trucks.

#### Rail

Transit and commuter railroads are assumed to be 100% electric. Intercity passenger and freight rail transport is assumed to operate with non-fossil hydrocarbon fuel. Note that the Northeast and Keystone corridors constitute the entire extent of electric inter-city rail in the US.

#### Agriculture, mining, and construction

These applications are usually omitted from studies of transportation energy. They are energy-intensive and operate in conditions inconducive to convenient charging. In the baseline model, it is assumed that they continue to operate with non-fossil hydrocarbon fuel.

#### Aviation

While a viable breakthrough in battery technology or H_2_ for regional flights cannot be ruled out, the baseline assumption is that all aircraft continue to operate with non-fossil hydrocarbon fuel.

#### Water transport

Although it is likely that some commercial shipping operations, such as ferries and tenders, could be electrified, the bulk of maritime fuel is used on open water. For simplicity, we assume that all maritime fuel will be non-fossil hydrocarbon.

#### Military use

Military energy use is dominated by aviation fuel for the Air Force and air arms of the Army and Navy (Crawford, 2019). The expense of bringing ground-vehicle fuel into operational theaters demands the highest possible energy density while field operations are not compatible with the logistical limits of all-electric vehicles. Therefore, all military fuel is assumed to be non-fossil hydrocarbon.

#### Lubricants

Because a large fraction of used lubricants is eventually recycled for fuel, we assume that lubricants are synthesized from non-fossil hydrocarbon.

## Results

### Energy

Data in AEO Projection supplemental tables can be rearranged into a table of the amounts and types of fuel used in each of the 51 economic subsectors (Table 2). The result of applying the fuel replacement assumptions previously described is shown in the top rows of Tables 4a and 4b. [Note that this is electricity delivered; electricity generated will be significantly higher owing to transmission and storage losses.] In the baseline case, the carbon-neutral version of the 2050 reference economy projected in the AEO will require nearly four times the electricity we use today (3882 billion kWh or bkWh), a result in good agreement with much richer models (Jacobson et al., 2015; Ram et al., 2019; Williams et al., 2012). This future economy will also require 1.04 billion metric tons of biomass annually. Although immense, this amount is less than the 1.19 billion dry tons projected in the baseline case of the Billion-Ton Report (Langholz et al., 2016). Transportation accounts for 45% of the incremental generation (EV: 20%, ebFuel synthesis: 25%).

**Table 4:** Use of electricity in the entire economy (a) and transportation only (b) in the 2050 baseline carbon-neutral economy in units of billion kilowatt-hour (bkWh)

(a) Total Economy	AEO	Conversion	ebFuel	eH_2_	eFuel	Total	Biomass	Liquid Fuel	H_2_
	bkWh	bkWh	bkWh	bkWh	bkWh	bkWh	btons	bgge	bkg
Baseline Case	4901	5905	3177	434		14,417	1.04	158	9
Minimum Biomass	4901	5905	1692	3175		15,624	0.56	84	65
Zero Biomass	4901	5905		3175	5737	19,719		84	65
(b) Transportation	AEO	Conversion	ebFuel	eH_2_	eFuel	Total	Biomass	Liquid Fuel	H_2_
	BkWh	BkWh	bkWh	bkWh	bkWh	bkWh	btons	bgge	bkg
Baseline Case	124	1902	2308			4334	0.76	115	
Minimum Biomass	124	1902	823	2742		5591	0.27	41	56
Zero Biomass	124	1902		2742	2790	7558		41	56
Max. Elect.	124	2695	1016			3918	0.33	51	
Base no TEV	124	1479	2932			4535	0.96	146	
Zero Bio. no TEV	124	1479		3952	2790	8345		41	81

Values do not include transmission and distribution losses. The column labeled AEO is electricity usage in the AEO reference case. The column labeled Conversion represents incremental consumption owing to the replacement of chemically fueled devices with their electric counterparts (electric vehicles, heat pumps, and so forth). Electricity for fuel synthesis is used to make H_2_ for high-heat stationary applications (eH_2_) and liquid hydrocarbon (ebFuel or eFuel) for mobile applications and bulk chemical feedstock.

The appetite for electricity is sensitive to what can be electrified directly, the choice of electro-fuel, and the efficiency of making that fuel. In the substitution model, it is easy to assess alternative scenarios. Because the baseline case demand for biomass is barely within expected availability and e-H_2_ in FCs is the next most electricity-efficient option, we examine the case where biomass-derived fuel is reserved for bulk chemicals, aviation, maritime, and military use while H_2_ is used in all other non-electrified transportation. In this “minimum biomass” case, biomass demand reduces to 270 million tons annually, with nearly half used as a bulk chemical feedstock, while total electricity demand increases by about 10% relative to the baseline case. As expected, approaches that do not take advantage of energy embodied in biomass require more electricity. A “zero-biomass” case with non-photosynthetic carbon collection would require nearly 50% more electricity than the baseline case.

Table 3b includes several other transportation scenarios of interest. In the “maximum electrification” case with direct electrification of all transport except aviation, maritime, and military usage, biomass needs are well within availability limits and transportation electricity needs are reduced by 10% compared to the baseline case. The “no TEV” cases illustrate the importance of electrifying long-distance bus and truck transport. Without truck electrification, biomass demand expands to 1.26 billion tons, roughly the expected availability in the high yield case of the Billion-Ton Report (1.52 billion tons). A “minimum biomass” case without TEV entails a 30% increase in electricity use. Without direct electrification via BEV and TEV, replacing all fossil fuel with ebFuel would require two billion tons per year (2 bt/yr) of biomass for transportation alone. Similarly, replacing that same amount of hydrocarbon fuel with eFuel would require nearly ten times today’s generating capacity.

### Cost

Estimates of the “cost of decarbonization” relative to hypothetical future prices of conventional energy commodities are of limited value. An expanding fossil fuel economy will drive energy costs upward while fossil fuels are not used in a carbon-neutral economy and so have no definable price. The value of such estimates lies in their ability to convince skeptical stakeholders that averting climate catastrophe will not bring economic catastrophe. For this purpose, it is essential that such estimates be simple and transparent.

While capital costs of generating and delivering non-fossil energy are reflected in techno-economic estimates of the unit costs of renewable fuels, the capital costs of fuel switching for specific applications decades in the future are more speculative. Conversion of aircraft, trucks, ships, and locomotives to non-fossil hydrocarbon fuel costs nothing. Replacing a gas stove or dryer with an electric equivalent costs little today. The incremental cost of a heat pump in lieu of a home gas furnace is significant today but is sure to decrease as volume grows. Similarly, BEV and FCEV are more expensive than their conventional counterparts, but there is growing optimism over the prospects for cost parity. It is yet to be seen whether a steel mill or cement plant fueled by H_2_ is more expensive to build than its fossil-fueled counterpart. To circumvent this problem, we make the gross simplification that by 2050 every energy-consuming device will have been replaced at least once owing to normal aging and obsolescence, and that the incremental capital cost of replacement with an “electrified” instead in lieu of a conventional version is insignificant. In this construct, incremental fuel cost is a surrogate for carbon mitigation cost.

Table 5 lists the commodity cost for fossil and non-fossil fuels used in this estimate. Table 6 shows the annual fuel cost for each of the scenarios in Table 4 as well as the incremental cost relative to the AEO reference case. With commodity costs as of this writing, the energy cost of decarbonization is small relative to the projected size of the 2050 economy. Even the electricity-intensive “no biomass” case costs less than 1% of GDP. This result is consistent with earlier studies and justifies the assumption that decarbonization will not have a negative impact on economic growth. More interestingly, the cost of decarbonization is negative in all transportation scenarios. The large discrepancy in total energy expenditure between the baseline case ($1217b) and the AEO ($2858b) is owing to higher projected fossil fuel prices in a larger, but conventional economy ($0.17/kWh for electricity, $6/gge for transportation fuel). Using these higher values would produce a significant negative cost for decarbonization in all scenarios. This halcyonic conclusion should be seen as an illustration of the fallacy of comparing the projected prices of obsolete commodities rather than a promise of huge cost savings by decarbonization.

**Table 5:** Cost of energy alternatives

	Unit Cost (2021)	b$/QBTU	Data Source
Electricity	$0.066/kWh	19.4	Statistica.com
NG	$4.00/1000cf.	3.9	EIA Natural Gas Weekly Update (EIA, 2021b)
Gasoline	$2.70/gge w/o tax	23.3	AAA Gas Prices
Thermal Coal	$1.92/million BTU	1.9	Statistica.com
e-H_2_	$3.00/kg	26.0	Kurtz et al. (2018)
ebFuel	$3.36/gge.	29.5	(Das and Saffron, 2019)
eFuel	$6.00/gge	50.0	Brynolf (2017), Connolly et al. (2014b)

Electricity and fossil fuel costs are as of January 2021. Non-fossil energy costs are future, high-volume estimates based on established technologies.

**Table 6:** Energy expenditures in billions of USD for several carbon-neutrality scenarios

(a) Total Economy	Electricity	ebFuel	eH2	eFuel	Total	Delta Cost
AEO Reference Case					$1,217	
Baseline Case	$715	$542	$27		$1,284	$67
Minimum Biomass	$715	$288	$197		$1,200	($17)
No Biomass	$715		$197	$489	$1,401	$184
(b) Transportation						
AEO Reference Case					$693	
Baseline Case	$134	$393			$527	($166)
Minimum Biomass	$134	$140	$170		$444	($249)
No Biomass	$134		$170	$238	$542	($151)
Max. Electrification	$192	$173			$365	($328)
Baseline no TEV	$106	$500			$606	($87)
Min Biomass no TEV	$106	$140	$245		$491	($202)
Zero Biomass no TEV	$106		$245	$238	$589	($104)

The AEO reference case ($1217b) does not match the total 2050 energy expenditure in the Outlook ($2858b) owing to much higher energy price projections for a much larger but still fossil-fueled economy.

## Discussion

This study was designed to convey the magnitude of the electrification challenge and implications of transportation technology choices. The single metric for this study is electricity usage. The results do not claim superior efficiency or lower cost of using biomass. Plants are quite inefficient converters of sunlight to stored energy and costs of eFuel and ebFuel at scale remain unknown. Nevertheless, given the immense differences between approaches, these results provide compelling guidance for policy design.

First, an aggressive program of direct electrification is the most powerful lever for reducing the demand for non-fossil electricity while keeping biomass requirements within realistic limits. For personal vehicles, such a strategy must assure the availability of a home charger for every BEV (including those without dedicated parking) and a network of direct-current fast chargers (DCFC) for intercity travel. This requirement does not mandate the installation of a charger entirely dedicated to each BEV, but rather seeks to assure access to at least one (possibly shared) charger. Research shows that the installation of chargers for opportunistic charging while parked is of little benefit when BEV range generally exceeds 200 km (Tamor, 2018). Even with such a strategy, the future electricity system will have at least three times the capacity of today’s and will require an equally capable transmission, distribution, and energy storage system. Only one-third of its output will go to traditional residential, commercial, and industrial customers. Another third will go to charging vehicle batteries, while the remainder will power immense fuel synthesis industries that do not exist today. Thus, we must anticipate that the future electricity system will operate very differently than it does today. The dominance of these new loads offers great flexibility and may reduce the need for grid storage in a renewable-rich energy system. It also brings into question the notion that renewable fuels can be synthesized with low-cost electricity at times of high electricity generation but low demand.

Second, even a highly electrified economy will need chemical fuels. Although personal vehicles can be mostly replaced with BEV, many transportation applications are difficult or impossible to electrify. In the baseline case with its high, but plausible penetration of BEV and TEV, 115 billion gge of transportation fuel will be needed each year. With chemical feedstocks included, the baseline case calls for 158 billion gge of hydrocarbon products per year. Note that the baseline case does not use hydrogen for transportation. The low biomass case replaces 74 billion gge of hydrocarbon transportation fuel with 56 billion gge of hydrogen.

Third, although biomass is a limited resource, studies suggest that there will be enough to meet the remaining demand for hydrocarbon fuel and chemical feedstocks. Synthesis of the requisite 158 billion gge of hydrocarbon will require 1.04 billion dry tons of biomass per year, which is less than the expected availability without an increase in yield, competition with food production, or conversion of natural areas to energy agriculture. This finding reinforces the essential role of carbon-efficient biomass conversion processes; synthesis of an equivalent amount of fuel by conventional fermentation would require twice as much biomass, well beyond projected availability. Even with carbon-efficient processes, the predicted biomass availability may fail to materialize or prove too expensive. If so, limiting biomass use to applications that demand the storability and energy density of liquid hydrocarbon—chemicals, aviation, mining, construction, agriculture, and military—will reduce biomass demand to 330 million dry tons annually. In the range of intermediate scenarios, achieving carbon-neutral transportation with the least electricity-intensive alternative, H_2_, will require the development of a parallel fuel industry capable of synthesizing and delivering as much as 51 billion kg of H_2_ per year. Although progress in battery technology has drawn attention away from FCEVs for personal vehicles, this finding reinforces the growing interest in the development of FC propulsion for large vehicles and potentially in aviation.

This study is consistent with others in concluding that the annual cost of decarbonization is at most on the order of 1% of GDP. This cost is the equivalent of having suffered a mild recession with one year of slow economic growth. The central challenge will be creating a regulatory environment that will enable financing of the greatest infrastructure build-out because the railroad boom following the Civil War or urban electrification a half-century later. Neither of these had a fixed deadline. Carbon neutrality by 2050 is (barely) enough time to remake our energy economy without stranding existing capital investments, but not enough to remake our urban landscape to minimize energy needs. Segmented, incremental policy approaches that do not recognize the merging of the stationary and mobile energy economies may impede this task. Tripling US electricity production, developing one, possibly two new fuel industries, and deploying the equipment that uses that energy in only 30 years will require an integrated and predictable regulatory and policy regime—including land use, environmental impacts, rights-of-way, and environmental justice—that reassures investors and consumers. We cannot predict technological progress and innovation, nor changes in energy usage, thirty years in advance. Therefore, policy must focus on the quantitative outcome, zero net fossil carbon emissions, while maximizing flexibility in achieving that outcome.

### Limitations of study

Studies such as this are no more reliable than the underlying projections of energy use in the US three decades in the future. Similarly, projections of progress in cost and efficiency of alternative fuel technologies, evolving consumer preferences, and social change should be treated with appropriate skepticism. Even if the NEMS forecasts are considered reliable, it is possible that the “front-loading” of large capital investment for renewable energy, followed later by lower operating costs, will have some impact on economic growth. As a difference between two very large numbers, the relative energy cost of carbon neutrality is sensitive to the projected costs of renewable electricity and fossil fuel. It is reasonable to expect that the cost of renewable energy will decline with scale while that of fossil fuel will rise with demand and resource depletion thus making carbon neutrality highly favorable even before considering the secondary benefits of abandoning fossil fuel. Despite the uncertainties as to which sectors of the economy will grow and by how much, the results are simple and robust: to attain carbon-neutrality while enjoying the comforts and choices we have today, we will need several times the electricity we use today.

## STAR★Methods

### Key resources table

**Table:** 

REAGENT or RESOURCE	SOURCE	IDENTIFIER
**Deposited data**		

Energy Use Projections	EIA Annual Energy Outlook 2021	https://www.eia.gov/outlooks/aeo/
Transportation Energy Data	Transportation Energy Data Book: Edition 39	https://tedb.ornl.gov/wp-content/uploads/2021/02/TEDB_Ed_39.pdf
Spreadsheet Model	Authors	https://zenodo.org/record/6406749/files/Tamor%20ISCIENCE-D-22-00543.xlsx?download=1

### Resource availability

#### Lead contact

Requests for further should be directed to the lead contact, Michael Tamor (mtamor@asu.edu).

#### Materials availability

No physical materials were developed by or for this project.

### Method details

Projected energy consumption for 51 economic activities in the Annual Energy Outlook (EIA, 2021a) were compiled in the model spreadsheet. Fossil fuels were replaced with non-fossil sources under the series of assumptions listed below. The total consumption of non-fossil energy (electricity and biomass) was then computed for each activity, economic segment, and the total economy. The fuel costs for the AEO forecast economy and the carbon-neutral version of that economy were computed based on the future cost estimates from sources listed in Table 5.

#### Assumptions

1. The petroleum refining industry has ceased to exist and any energy needs for replacement products are included in the energy cost of synthesizing replacement fuels.
2. With no synthetic natural gas in our model and transportation energy already included in the efficiency of H_2_ synthesis and delivery, pipeline fuel that drives pumps and heaters in the national pipeline system is eliminated.
3. Lease and plant fuel for petroleum and natural gas exploration is eliminated entirely.
4. Petroleum-derived paving tar is replaced with an unspecified non-fossil product.
5. Residential and commercial fossil fuel usage is completely replaced by electricity via two pathways:
  a. Replacement of natural gas- or oil-fired cooking equipment with electric assuming resistive heating.
  b. Replacement of natural gas- or oil-fired space and water heating with electric heat pumps with a coefficient of performance of 3.
6. Hydrocarbon feedstock for the bulk chemical industry is derived from biomass or air-captured CO_2_.
7. The industrial manufacturing sector is entirely electrified except for cement and lime, aluminum, glass, and iron and steel industries. Fossil fuel for these four industrial sectors is replaced with H_2_ from non-fossil sources.
8. Natural gas, diesel, and coal used in the nonmanufacturing industrial sector (mining, construction, and agriculture) are replaced by electricity. Use of petroleum distillates, mainly diesel fuel, mainly for mobile equipment is treated as a subsector of transportation.

## Data Availability

The Excel spreadsheet developed for this study is available for download from GitHub (https://zenodo.org/record/6406749/files/Tamor%20ISCIENCE-D-22-00543.xlsx?download=1). The spreadsheet includes the AEO energy use projections for 2050 (also shown in Table 2), a coefficient of performance for the electrified replacement for combustion equipment in each activity: an actual coefficient of performance (COP) for heat pumps, a COP of 1.0 for replacing combustion heat with resistive heat, and an electrification fraction indicating the fraction transportation energy that may be provided by electricity via a battery electric vehicle (BEV) or tethered electric vehicle (TEV). The spreadsheet includes a table of conversion factors, a table of costs for fossil and renewable energy, a set of flags to select scenarios, and a location to include a user-defined set of electrification factors. Any additional information required to reanalyze the data reported in this paper is available from the lead contact upon request.
